# Tuneable complementary metamaterial structures based on graphene for single and multiple transparency windows

**DOI:** 10.1038/srep06128

**Published:** 2014-08-22

**Authors:** Jun Ding, Bayaner Arigong, Han Ren, Mi Zhou, Jin Shao, Meng Lu, Yang Chai, Yuankun Lin, Hualiang Zhang

**Affiliations:** 1 Electrical Engineering Department, University of North Texas, 3940 N. Elm St., Denton, TX 76207; 2 Physics Department, University of North Texas, Denton, TX 76203; 3 Department of Applied Physics, The Hong Kong Polytechnic University, Hong Kong; 4 Department of Electrical and Computer Engineering, Iowa State University, Ames, IA 50011

## Abstract

Novel graphene-based tunable plasmonic metamaterials featuring single and multiple transparency windows are numerically studied in this paper. The designed structures consist of a graphene layer perforated with quadrupole slot structures and dolmen-like slot structures printed on a substrate. Specifically, the graphene-based quadrupole slot structure can realize a single transparency window, which is achieved without breaking the structure symmetry. Further investigations have shown that the single transparency window in the proposed quadrupole slot structure is more likely originated from the quantum effect of Autler-Townes splitting. Then, by introducing a dipole slot to the quadrupole slot structure to form the dolmen-like slot structure, an additional transmission dip could occur in the transmission spectrum, thus, a multiple-transparency-window system can be achieved (for the first time for graphene-based devices). More importantly, the transparency windows for both the quadrupole slot and the dolmen-like slot structures can be dynamically controlled over a broad frequency range by varying the Fermi energy levels of the graphene layer (through electrostatic gating). The proposed slot metamaterial structures with tunable single and multiple transparency windows could find potential applications in many areas such as multiple-wavelength slow-light devices, active plasmonic switching, and optical sensing.

Both quantum effects of Autler-Townes splitting (ATS) and electromagnetically induced transparency (EIT) can generate a transparency window in the transmission spectrum, which has many important applications in slow light devices^1^, bio-sensing^2^, plasmonic switching^3^, plasmon rulers^4^, surface enhanced Raman scattering (SERS)^5^. Although ATS and EIT share similar spectra lineshapes, and can be understood by a coupled two-oscillator system, even crossover could happen between EIT and ATS under some situations^6^,^7^, their origins are different^8^: in EIT the transparency window is a quantum destructive interference between the energy transferred from an initial state to a final state via different pathways, whereas in ATS it is due to the strong-coupling induced splitting of resonant modes. In the following, a system that has a transparency window but is not originated from EIT will be called an EIT-like system. The EIT and EIT-like systems have been widely studied in metamaterial structures including nanoparticle structures^9^,^10^,^11^,^12^ and nanoslot structures^13^,^14^,^15^.

In general, the ability to actively control the resonant elements of the metamaterial structures is very attractive since it can be utilized to dynamically control their optical response and potentially expand the range of their applications further. So far, most of the transparency windows observed in metamaterial structures have been realized at a fixed working wavelength. In these cases, the resonance has to be tuned to different working wavelengths by carefully reconstructing the geometries or modifying the supporting substrates, which are difficult (if not impossible) to achieve once the devices are fabricated. To address this issue, many approaches to achieve dynamic tuning of the transparency windows have emerged by integrating metamaterials with materials with tuneable permittivity, such as nonlinear media^16^, liquid crystals^17^, and phase-change materials^18^. Especially graphene, a single layer of carbon atoms gathered in a honeycomb lattice, has been recently investigated for nanoscale micro-electronic integrated circuits and plasmonic devices^19^ since it exhibits many unique physical features. Particularly, it is found that graphene appears to be a good candidate for designing and engineering tuneable devices because its conductivity can be controlled by shifting the Fermi energy levels, which may be potentially tuned from −1 to 1 eV by chemical doping^20^ or electrostatic gating^21^. Moreover, graphene can strongly interact with light over a wide frequency range from the THz to infrared frequencies. It is especially suited for the mid-IR frequency range because of the combination of its strong plasmonic response and negligible loss. This allows for the creation of surface plasmon based devices that can be turned on and off effectively and conveniently or tuned to work at different frequencies. Meanwhile, along with the extensively studies of systems with single transparency window in the past few years, systems with multiple transparency windows have gradually gained much more attention^18^,^22^,^23^,^24^,^25^,^26^,^27^ as well, which may find more flexible and critical applications for multi-wavelength SERS and bio-sensing.

In this paper, we propose to dynamically manipulate the transparency windows by using planar complementary metamaterial structures based on graphene. Both single and double transparency windows are possible to be achieved by adjusting the coupling strength between the proposed structure elements. We started by studying the properties of a single layer graphene perforated with two parallel paired slots, i.e., a quadrupole slot resonant structure, in which a transparency window occurred between two transmission dips. By varying the distance between the two slots and examining the spectrum responses, we found that the transparency window is more likely caused by the ATS rather than the conventional EIT. Then, by placing a third slot, i.e., a dipole slot resonant structure, with the longer length perpendicular to the paired slot to form a dolmen-like structure, an additional transmission dip is introduced, thus, a system with two transparency windows can be realized. In addition, both transparency windows of the quadrupole slot structure and the dolmen-like slot structure can be dynamically tuned by adjusting the bias voltage (electrostatic gating) applied to the graphene layer. The presented complementary graphene-based metamaterial structures with multiple tuneable transparency windows could open up new opportunities for potential applications in tuneable multi-wavelength slow light devices and optical sensors.

## Results

### Proposed Designs

**Figure 1:**
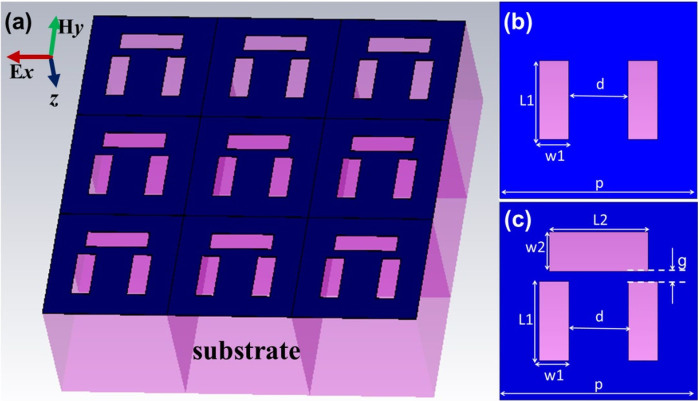
(a) Schematic of the complementary metamaterial design and the incident light polarization configuration; (b) unit cell of the quadrupole slot structure, L1 = 80 nm, w1 = 30 nm, and p = 200 nm; (c) unit cell of the dolmen-like slot structure L1 = 80 nm, w1 = w2 = 30 nm, d = 60 nm, and p = 200 nm.

Figure 1(a) schematically depicts the array of periodically arranged complementary dolmen structure (i.e., the dolmen-like slot structure under investigation), which is composed of a graphene layer perforated with three slots printed on a substrate. The graphene layer and the substrate are illustrated in blue and pink, respectively. The dielectric constant of the substrate is 2.1. Figure 1 (b) – (c) display the top view of a unit cell of a quadrupole slot structure, i.e., two parallel paired slots, and a dolmen-like slot structure, respectively. The complementary dolmen-like structure shown in Fig. 1 (c) is composed of a single slot (i.e., a dipole slot structure) on the top and a quadrupole slot structure at the bottom. The length and width of the slot in the quadrupole (dipole) structure are L1 (L2) and w1 (w2), respectively. The horizontal distance between the two slots in the quadrupole resonant structure is noted as *d*, and the vertical gap distance between the two resonant slot structures is noted as *g*. The details of the geometrical parameters of proposed structures are shown in the caption of Fig. 1. The array periodicities in both *x* and *y* directions are p = 200 nm. The designed devices are investigated in the mid-infrared frequency range at around 25~50 THz.

All the designed graphene-based tuneable metamaterial structures with EIT-like transparency windows are numerically studied by utilizing the commercial package CST Microwave Studio. The optical conductivity σ of graphene consists of the Drude (intraband) and interband contributions and is related to the Fermi energy or chemical potential *μ_c_*^28^, through 
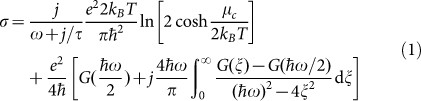
where 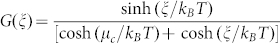, *e*, *k_B_* and ħ are the charge of an electron, the Boltzmann constant, and the reduced Planck constant, respectively. We assume an electron scattering lifetime τ = 1 ps, and a room temperature of *T* = 300 K in equation (1). In the mid-IR frequency range, such a conductivity form indicates strong plasmonic response. As can be seen from equation (1), changing the Fermi energy *μ_c_* enables the control of the propagation characteristics of the graphene plasmons.

### Quadrupole slot resonant structure

**Figure 2:**
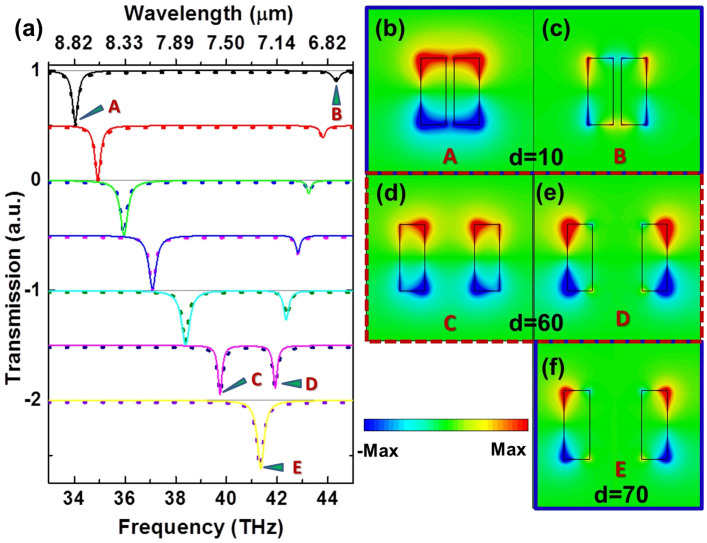
(a) Simulated (dot line) and fitted (solid line) transmission spectra for the quadrupole slot structure with different distances between the two slots, with L1 = 80 nm and w1 = 30 nm; (b)(c) *Hz* distributions at two transmission dips (A and B) for *d* = 10 nm, respectively; (d)(e) *Hz* distributions at two transmission dips (C and D) for *d* = 60 nm, respectively; (f) *Hz* distribution at the only transmission dip (E) for *d* = 70 nm.

Before studying the complementary dolmen-like structure as shown in Fig. 1(a) and (c), we started by investigating the quadrupole slot resonant structure shown in Fig. 1(b). It is known that the quadrupole nanoparticles, i.e., the counterpart of the quadrupole slot structure, are non-radiative under the *x*-polarized incident waves (see Fig. 1 (a)), which is usually deemed as the dark mode or sub-radiant mode in an EIT system. However, in the designed complementary structure based on graphene (as shown in Fig. 1 (b)), we observed a transparency window with two transmission dips in the transmission spectrum under the *x*-polarized excitation (the results are shown in Fig. 2). In order to find out the origin of the occurrence of the transparency window in the quadrupole slot structure, we conducted a parametric study by varying the separation distance *d* between the two slots from 10 nm to 70 nm. The calculated (dot line) and fitted (solid line) transmission spectra are displayed in Fig. 2(a). It is noticed that the coupling strength is increased (the splitting/separation between the two transmission dips, i.e., resonant modes, is increased) when the distance between the two slots *d* is decreased (from the bottom to the top curve). At *d* = 70 nm, there is only one resonant mode due to the periodicity of the plasmonic structure in the *x* direction. When the distance *d* is appropriate, e.g., *d* = 60 nm, a narrow transparency window will appear between two sharp transmission dips. To better understand the occurrence of the transparency window, the distributions of magnetic field normal to the *x*-*y* plane, i.e., the *Hz* at the interface of the graphene layer and substrate are computed and plotted in Fig. 2(b)~(f) at different transmission dips: the transmission dips A and B for *d* = 10 nm are plotted in Fig. 2(b) and (c), respectively; the transmission dips C and D for *d* = 60 nm are plotted in Fig. 2(d) and (e), respectively; the only transmission dip E for *d* = 70 nm is plotted in Fig. 2(f). As can be seen from Fig. 2(c), (e) and (f), the resonant modes at the transmission dips B, D, and E for different *d* have similar *Hz* distributions despite of the intensity difference, which indicates that this resonance comes from the same origin. As *d* decreases, a second resonant mode is induced as shown in Fig. 2(b) and (d) for transmission dips A and C, respectively. These *Hz* distributions (in Fig. 2 (b) and (d)) show a dipole-like resonant mode when the two slots are treated as a whole. It is also observed that the closer the two slots are, the stronger the resonant mode is. Overall, the two slots in the proposed quadrupole slot structure could be deemed as two oscillators with the similar Q^8^, and the transparency window is more likely caused by the strong-coupling induced splitting of the resonance mode, rather than the destructive inference between the bright and dark modes in a typical EIT system, which is further discussed by the fitted parameters from the analytical fitting of the transmission spectra in the following.

**Figure 3:**
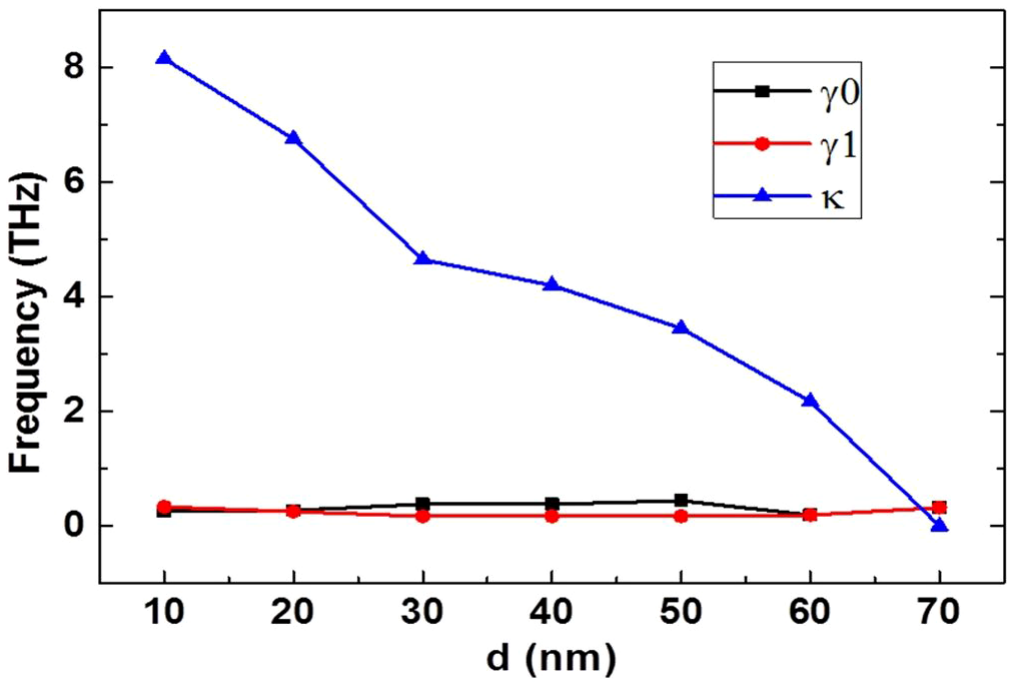
Extracted numerical damping and coupling parameters as a function of separation *d* between the two slots. Values of *γ*_0_, *γ*_1_ and *κ* are extracted by fitting the numerical transmission spectra through equation (2).

The above behaviors can be further understood by the classical coupled two-oscillator systems^8^,^29^,^30^

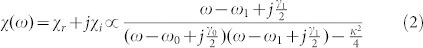
where *γ*_0_ and *γ*_1_ are the damping factors in the two oscillators, which represent losses, *κ* is the coupling efficient between the two oscillators. The equation (2) was derived with the approximation *ω*~*ω*_0.1_≫*κ*. The imaginary part of susceptibility χ is proportional to the energy dissipation in the system, thus, we can obtain the relationship between the transmission and χ through *T*(*ω*) = 1−*gχ_i_*(*ω*), where *g* is a geometric parameter indicating the coupling strength between the resonant mode and the incident electromagnetic field. The analytical fittings of the simulated transmission spectra for different *d* are shown in Fig. 2(a) (solid lines). As can be seen from Fig. 2(a), these two sets of results agree very well with each other. The fitted parameters for different separation *d* are plotted in Fig. 3.

In Fig. 3, when d increases, the coupling coefficient *κ* between the two oscillators decreases almost linearly from *κ* = 8.15 THz for *d* = 10 nm to κ = 0 for *d* = 70 nm, which confirms the assumption *ω*~*ω*_0.1_≫*κ* because *ω*_0,1_ are around 40 THz. The *κ* = 0 could be deemed as a degenerated case due to the symmetry in the array. It is also noticed in Fig. 3 that *κ≫γ*_0_*~γ*_1_ except for *d* = 70 nm, indicating that the two oscillators are in the strong-coupling regime^8^,^31^. From Fig. 2 (a) (by watching the evolution of the transparency window features), we observed that a second resonant mode is induced by the graphene-based quadrupole slot structure, and the splitting/separation between the two resonant modes gets larger as the coupling strength between the two oscillators gets stronger. Together with the confirmation from the fitted parameters as shown in Fig. 3 that the two oscillators are in the strong coupling regime, we can deduce that the transparency window in the quadrupole slot structure is more likely caused by the ATS rather than the traditional EIT.

### Double transparency windows

**Figure 4:**
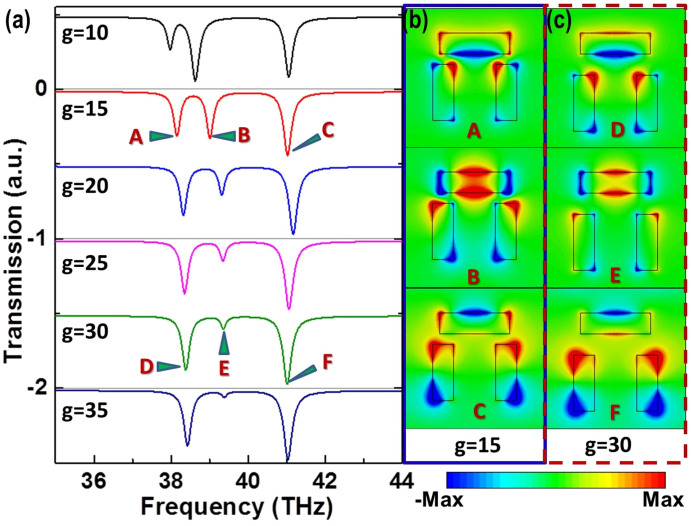
(a) Transmission spectra for the complementary dolmen-like structure with different gap distances between the two slot structures, L1 = 80 nm, L2 = 100 nm, w1 = w2 = 30 nm and d = 60 nm; (b) the calculated *Hz* distributions at three dips (A, B, and C) for g = 15 nm; (c) the calculated *Hz* distributions at three dips (D, E, and F) for g = 30 nm.

Next we investigate a multiple-transparency-window system by introducing a third slot perpendicular to the quadrupole slot structure shown in Fig. 1(c), which forms a dolmen-like slot structure. In practice, a system with multiple transparency windows is highly desirable in many applications. For an EIT based system, it usually consists of more dark or bright modes or takes the nested structures putting several close enough resonances together to form the multiple transparency windows. Here, we demonstrated that two transparency windows can be achieved in the designed complementary dolmen-like structure as shown in Fig. 1(c). Figure 4(a) displays the transmission spectra of the dolmen-like structure with different gap distance *g* between the two slot-type resonant structures (i.e., the dipole slot and the quadrupole slot). It is observed that a third transmission dip (located at the center of the two original dips) gradually becomes prominent as *g* decreases, while both of the two original transmission dips generated by the quadrupole slot structure remain almost unchanged. The distributions of the magnetic field (*Hz*) at the interface of graphene layer and substrate at the three transmission dips are plotted in Fig. 4(b) and (c) for *g* = 15 nm and *g* = 30 nm, respectively. Once again, the field distributions are very similar to each other at each corresponding dip for different *g* despite of intensity difference. It is noted that the field intensity is much larger for the middle dip (B and E as labeled in Fig. 4(a)) with *g* = 15 nm than *g* = 30 nm, indicating a stronger interaction between the two slot-type resonant structures and demonstrating a deeper dip in the transmission spectrum. As a result, two noticeable transparency windows can be obtained with appropriate gap distances, such as 10 nm< *g* <20 nm.

**Figure 5:**
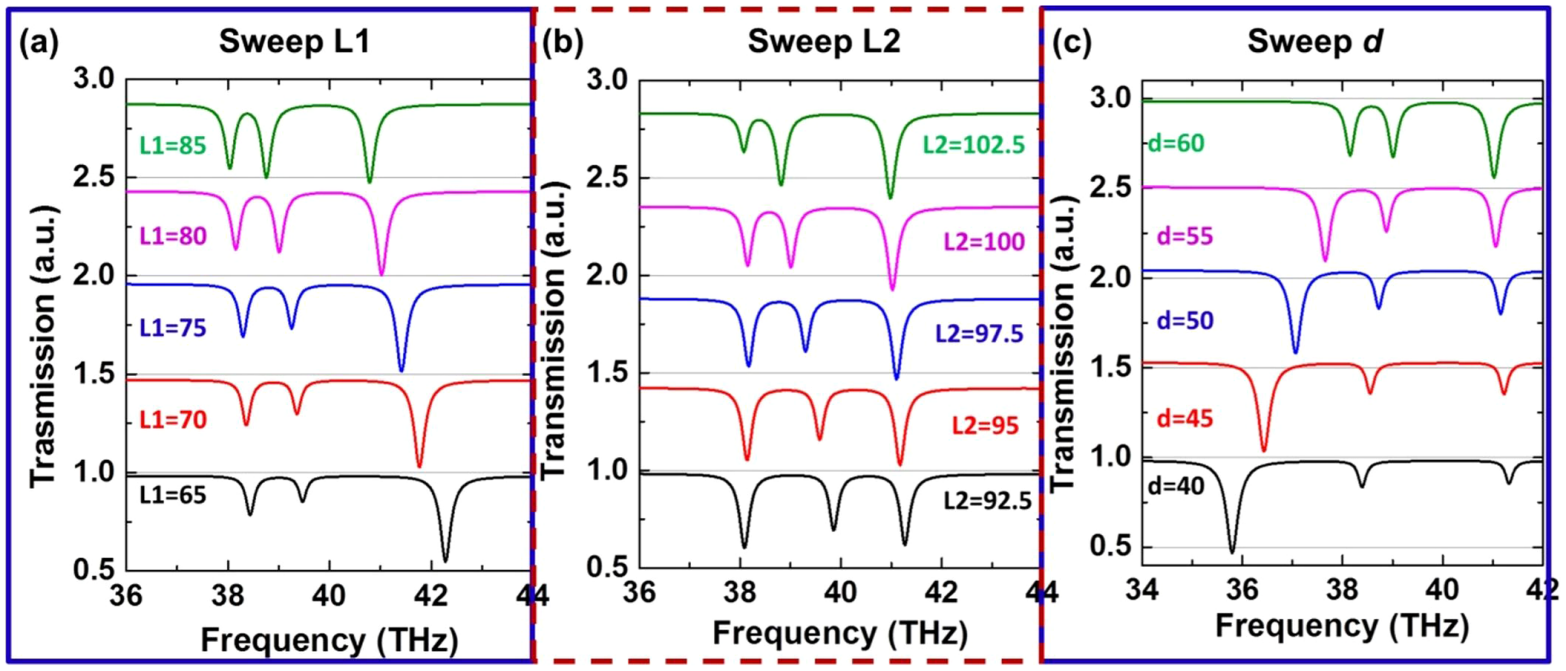
The simulated transmission spectra for (a) different L1 with L2 = 100 nm and d = 60 nm, (b) different L2 with L1 = 80 nm and d = 60 nm, and (c) different d with L1 = 80 nm and L2 = 100 nm.

As mentioned earlier, the middle dip in the transmission spectrum is mainly introduced by the additional dipole slot, while the other two dips by the quadrupole slot, which can be observed from the *Hz* distributions in Fig. 4 (b) and (c). Although the couplings between the slots are complicated, it can be still observed that the dipole slot is excited at transmission dips B and E, while the quadrupole slot mainly contributes to transmission dips A, C, D, and F. Now we conduct parametric studies to further investigate the effect of the geometrical parameters to the transparency windows between the three transmission dips. Throughout the parametric studies, the width of all slots is fixed at w1 = w2 = 30 nm, and gap *g* = 15 nm. In the parametric studies, one of the three parameters, i.e., L1, L2, and *d*, is varied, while the other two are fixed. The simulated transmission spectra for different L1, L2, and *d* are demonstrated in Fig. 5 (a), (b), and (c), respectively. In Fig. 5(a), by decreasing the length of the quadrupole slot (L1) from top to bottom, it is noticed that the first two dips at lower frequencies are slightly blue-shifted and the depths of these two dips become smaller, while the third dip at the highest frequency is obviously blue-shifted and the depth of this dip becomes larger, indicating a stronger resonance. This can be explained by the dipole-like resonance of the quadrupole slot structures, as referred to the *Hz* distributions at transmission dips at C and F in Fig. 4. In Fig. 5(b), by decreasing the length of the dipole slot (L2) from top to bottom, it is observed that the first and third dips remain almost unchanged, while the middle dip, i.e., the second dip, is blue-shifted prominently. Once again, this can be explained by the strong resonance of the dipole slot structure referring to the *Hz* distributions at transmission dips B and E in Fig. 4. In Fig. 5(c), with the decrease of the separation of the two slots in the quadrupole slot structure, it is observed that the second and third dips are slightly red-shifted and blue-shifted, respectively, and the depths of these two dips become smaller, while the first dip is red-shifted greatly and the depth of it becomes larger. This phenomenon is very similar to the single quadrupole slot structure studied previously without the dipole slot structure. Although the coupling mechanism is complicated in the dolmen-like slot structure, it is found that the positions of transmission dips in the transmission spectra, and thus the multiple transmission transparency windows induced by this structure can be manipulated by varying the lengths of the slots or the separation between the two slots in the quadrupole slot structure.

### Tuneability

**Figure 6:**
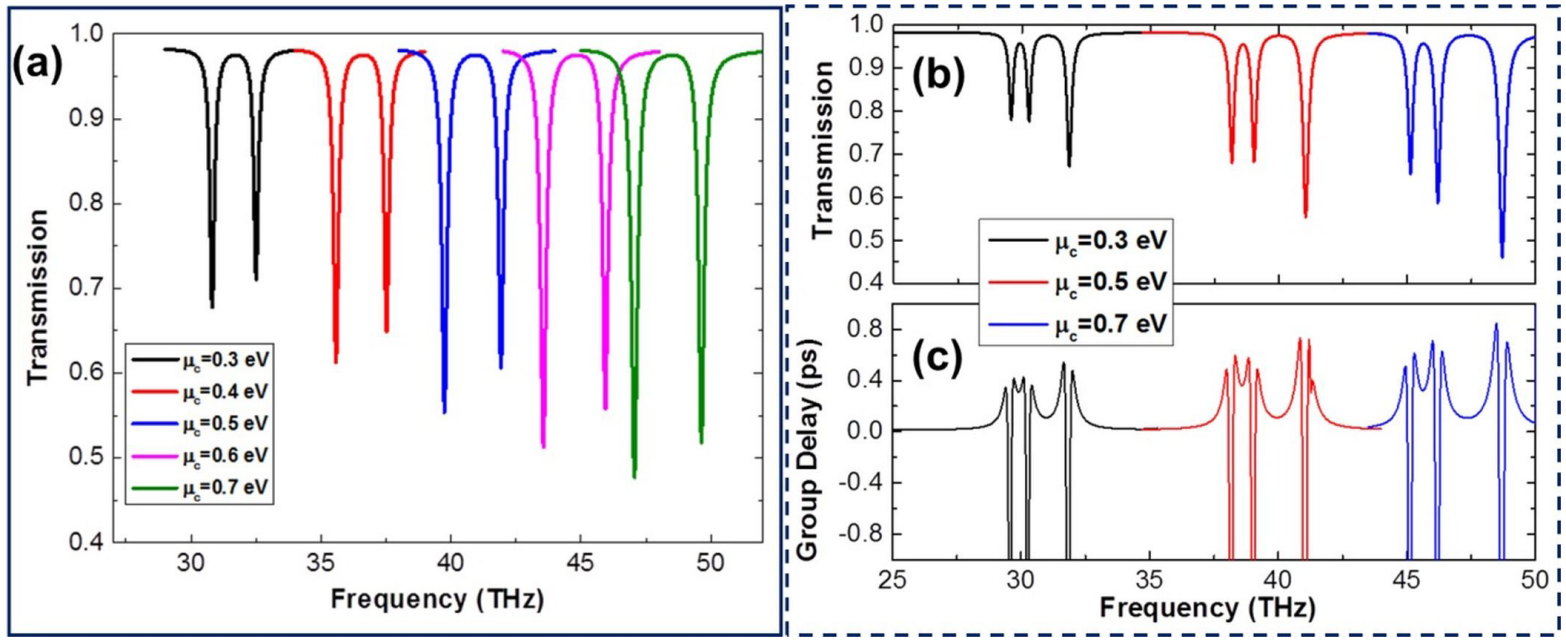
(a) and (b) Transmission spectra for the quadrupole slot structure and the complementary dolmen-like structure at different Fermi energy levels, respectively; L1 = 80 nm, L2 = 100 nm, w1 = w2 = 30 nm, *d* = 60 nm, and *g* = 15 nm; (c) the corresponding group delay for the complementary dolmen-like structure.

Furthermore and most importantly, one of the major advantages of the designed structures with graphene (as compared with noble-metal-based structures) is the tuneability, which can be achieved by chemical doping or electrostatic gating. Specifically, the optical response of the designed structures can be changed by applying different bias voltages so that they can work at different wavelengths (frequencies) without re-optimizing or reconstructing the physical structure. This is highly desirable in many practical applications since it is very difficult to change the physical structure after fabrication. Moreover, the proposed metamaterial structures are perforated on a graphene layer which is electrically connected^32^, therefore, it can be conveniently tuned by the means of electrostatic gating with just one biasing control (Note: the conventional graphene structures such as nanostrips need multiple biasing controls, which are not convenient for practical applications). To demonstrate the wavelength tuneability of the designed graphene-based complementary metamaterial structures, we plotted in Fig. 6(a) and (b) the transmission spectra of the quadrupole slot structure and the dolmen-like slot structure at different graphene Fermi energy levels, respectively. It is clearly seen that the transparency windows (single and double windows) can be shifted over a fairly broad range in the investigated frequency regime by merely varying *μ_c_*, which is difficult to be achieved in metal-based systems and highly desirable for practical applications. Based on the tuneable transparency windows, switches or modulators can be designed to operate in the investigated frequency regime. For example, considering the transmission spectra in Fig. 6(a), the transmissions for *μ_c_* = 0.5 *eV* and *μ_c_* = 0.6 *eV* at f = 39.75 THz are 0.554 and 0.982, respectively, which indicates that the transmission could be switched between 55.4% and 98.2% by simply varying *μ_c_* with a variation of 0.1 *eV*. Thus, a transmission magnitude modulation depth of 43% could be achieved through a small variation of *μ_c_* (0.1 *eV*). With a large variation of *μ_c_*, the level of modulation depth can be further increased. Also, it is noted that the resonant strength of both structures is enhanced and blue shifted as the Fermi energy increases. This behavior can be interpreted through the resonance condition. It is know that the wave vector of surface plasmons along the graphene layer satisfies 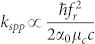
33, where 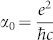 is the fine structure constant, and *f_r_* is the resonant frequency. Thus, the resonant frequency *f_r_* can be approximately described as a function of Fermi energy 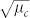. Therefore, the resonances and transparency windows of the two designed slot structures can be tuned by merely varying the Fermi energy of graphene with fixed geometrical parameters. In addition, in Fig. 6(c), we also displayed the corresponding group delays for the dolmen-like slot structure at different graphene Fermi energy. Positive and negative group delays correspond to slow and fast light, respectively. As can be seen from Fig. 6(c), it has large positive group delays in the vicinity of the peaks of the transparency windows, indicating slow light effect. Obviously, the group delays could be tuned at different Fermi energy as well as the transmission spectra. Therefore, we could achieve the capability of controlling the group delay by varying the Fermi energy. This property along with the large group delay of the designed devices has made them attractive for slow light applications including ultra-sensitive bio-sensors and telecommunications^34^,^35^, capable of covering multiple frequencies.

## Discussion

In conclusion, we have numerically demonstrated complementary metamaterial structures based on graphene to achieve single or multiple transparency windows, which can be dynamically controlled. A transparency window between two transmission dips can be realized by the graphene layer perforated with a quadrupole slot structure. When the distance *d* between the two parallel paired slots is gradually increased, the two transmission dips are gradually separated from each other and the *z*-component of magnetic field *Hz* at each corresponding dip have similar distributions for different *d*, which indicates the transparency window is likely caused by ATS, i.e., the strong-coupling induced splitting of the resonance modes. In addition, by introducing closely a dipole slot structure to the quadrupole slot structure to form a dolmen-like slot structure, a third transmission dip appears in the transmission spectrum, resulting in multiple transparency windows. The additional third transmission dip becomes prominent when the two slot resonant structures are close enough to each other. Through parametric studies, we notice that the first transmission dip in the transmission spectra of the dolmen-like slot structure is mainly contributed from the separation between the two slots in the quadrupole slot structure, the second transmission dip from the length of the dipole slot structure, while the third dip from the length of the quadrupole slot structure. Furthermore, the transparency windows for both complementary quadrupole and dolmen-like structures can be dynamically tuned by varying the Fermi energy applying on the graphene layer, which could not be achieved by the metal based metamaterials. The new complementary metamaterial structures based on graphene featuring single and multiple tuneable transparency windows may open up new avenues for designing tuneable multiple-wavelength slow light devices, plasmonic switches, and optical sensors.

## Methods

For the numerical calculations, we used the commercial package CST Microwave Studio 2014 by means of Finite Element Method. A frequency-domain solver is adopted with unit-cell boundary conditions in the *x*-*y* plane (as shown in Fig. 1) and Floquet ports in the *z* direction for terminating the whole domain. The incident light is normal to the *x*-*y* plane with E field polarized in *x*-direction. The simulation domain is meshed by tetrahedral, and the adaptive tetrahedral mesh refinement in the solving process is utilized to ensure convergent solutions.

For modeling the one-atom thick graphene layer, we treated the graphene as an extreme thin layer with an effective dielectric constant directly related to the conductivity^36^. After obtaining the conductivity of the graphene layer from Equation (1), the dielectric constant of graphene can be calculated through 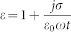, where *t* is the thickness of graphene in modeling and simulation. Typical values for *t* could be 0.35 nm (the approximate value of the diameter of an atom), 0.7 nm, or 1 nm. Although these typical values make no difference for the final result, the smaller value of *t* requires more computational resource, we adopted *t* = 1 nm throughout our simulations.

## Author Contributions

J.D. and H.Z. conceived the idea. J.D. designed the devices and performed numerical simulations. H.Z. supervised the research. J.D. and H.Z. co-wrote the manuscript. B.A., H.R., M.Z., J.S., M.L., Y.C., and Y.L. edited and reviewed the manuscript.
